# Biological Roles of Melanin and Natural Product-Derived Approaches for Its Modulation

**DOI:** 10.3390/ijms27020653

**Published:** 2026-01-08

**Authors:** Sunghyun Hong, Hanbin Lim, Do-Hee Kim

**Affiliations:** Department of Chemistry, Kyonggi University, Suwon 16227, Gyeonggi-do, Republic of Korea

**Keywords:** melanin, tyrosinase, natural product, melanogenesis, skin

## Abstract

Melanin produced in melanocytes contributes to photoprotection, oxidative stress reduction, immune regulation, and epidermal homeostasis, while its dysregulation underlies diverse pigmentary disorders. Natural products modulate melanogenesis by regulating tyrosinase activity, intracellular signaling pathways such as extracellular signal-regulated kinase/mitogen-activated protein kinase (ERK/MAPK) and cyclicAMP/protein kinase A/cAMP response element-binding protein (cAMP/PKA/CREB), and cellular redox balance. Anti-melanogenic effects have been reported for various fruit-derived phytochemicals, ginseng-based metabolites, and plant polyphenols, which act through direct enzymatic inhibition, suppression of melanoenic signaling, modulation of melanosome dynamics, and antioxidant or anti-inflammatory activities. Advances in delivery systems, including nano- and microencapsulation platforms, further enhance the stability and topical bioavailability of these compounds. In contrast, certain methoxylated flavonoids and phenolic constituents can stimulate pigmentation by sustaining melanogenic signaling and promoting microphthalmia-associated transcription factor (MITF)-driven transcription, emphasizing the context-dependent and bidirectional influence of natural substances on pigmentation outcomes. Collectively, these findings highlight the therapeutic potential of natural product-based modulators of melanogenesis while underscoring the need for mechanistic clarification, safety evaluation, and translational studies to ensure effective and controlled pigmentation management. This review summarizes the biological functions of melanin and examines natural strategies for regulating pigmentation.

## 1. Introduction

Melanin is a widely distributed biological pigment found in multiple human tissues, including the skin, hair, and eyes, where it determines color variation and supports essential physiological processes [1]. Beyond its role in pigmentation, melanin protects against ultraviolet (UV)-radiation-induced cellular damage, contributes to cutaneous immune regulation, and participates in neurobiological signaling. Among its functions, eumelanin-mediated photoprotection is particularly important, enabling the skin to mitigate UV-driven oxidative stress and preserves genomic integrity [2,3,4].

Melanin is synthesized by melanocytes, which are specialized pigment-producing cells located primarily in the epidermis and hair follicles. With these cells, melanin is generated in membrane-bound organelles known as melanosomes, which undergo a maturation process before being transferred to surrounding keratinocytes [5]. Three major forms of melanin have been identified, eumelanin, pheomelanin, and neuromelanin, with each characterized by distinct physicochemical properties and biological roles. Eumelanin, the predominant type, imparts brown to black coloration and provides robust protection against UV radiation. Pheomelanin, which yields red to yellow pigments, offers weaker photoprotection and is more susceptible to UV-induced oxidative damage. Neuromelanin is localized in specific regions of the brain, where it contributes to metal ion sequestration and neuronal homeostasis [6].

Melanocytes also act as immunomodulatory cells by releasing pro-inflammatory cytokines, chemokines, and damage-associated molecular patterns that help maintain cutaneous immune homeostasis [7,8]. Their immunological functions are continuously modulated by environmental and physiological stimuli such as UV radiation, alterations in the skin microbiome, and oxidative stress [9,10,11]. The extent and characteristics of these immunological activities vary among skin disorders, reflecting disease-specific regulatory dynamics [12]. In addition, alterations in melanin levels and microbiome composition have been associated with multiple neurodegenerative disorders, suggesting broader systemic implication [13].

Dietary and topical factors have been investigated for their influence on melanogenesis and skin aging. Fruits contain abundant antioxidants, anti-inflammatory agents, vitamins, minerals, and polyphenols, positioning them as valuable contributors to skin antioxidation, brightening, and wrinkle reduction. These properties have prompted growing interest in leveraging fruits and fruit-derived extracts to alleviate visible signs of skin aging [14]. Numerous reports highlight their capacity to improve moisturization, diminish pigmentation, and reduce wrinkles, thereby supporting youthful appearance. However, despite widely recognized skin care benefits, systematic research characterizing the overall anti-aging mechanisms of fruit-derived components remains limited [15,16]. Research on plant-derived natural products and their regulatory effects on melanin biosynthesis is extensive; however, investigations specifically addressing fruit-based sources remain relatively limited. This situation emphasizes the need for comprehensive and detailed analytical studies to clarify the melanogenesis-modulating potential of fruit-derived bioactive compounds.

This study aims to delineate the multifaceted biological roles of melanin, examine its contribution to melanoma initiation and progression, and evaluate natural products reported to modulate melanogenesis, with a particular emphasis on fruit-derived bioactive compounds as key sources of melanogenesis-regulating agents. These insights provide the foundation for developing melanogenesis-targeted strategies with therapeutic and cosmetic applications.

## 2. The Metabolism of Melanin Synthesis

Melanin biosynthesis is initiated from the conditionally nonessential amino acid tyrosine, which is obtained either through dietary intake or via endogenous synthesis from the essential amino acid phenylalanine. This conversion is catalyzed by phenylalanine hydroxylase, which hydroxylates the aromatic ring of phenylalanine to generate tyrosine [17]. Melanogenesis is activated by α-melanocyte-stimulating hormone (α-MSH), which increases tyrosine availability for pigment synthesis by facilitating its uptake into melanocytes and, more specifically, into melanosomes [18]. Upon binding its receptor, α-MSH triggers the cyclic adenosine monophosphate (cAMP)-protein kinase A (PKA)-cAMP response element-binding protein (CREB) signaling pathway, resulting in enhanced transcription of microphthalmia-associated transcription factor (MITF), which is the central regulator of melanocyte differentiation and pigment-related gene expression. MITF subsequently induces the expression of the key melanogenic enzymes tyrosinase (TYR), TYRP1, and TYRP2, thereby promoting melanin synthesis [19,20,21]. In addition, MITF upregulates the amino acid transporter SLC7A5 (LAT1), which contributes to intracellular tyrosine uptake and supports continued pigment production [22].

**Figure 1:**
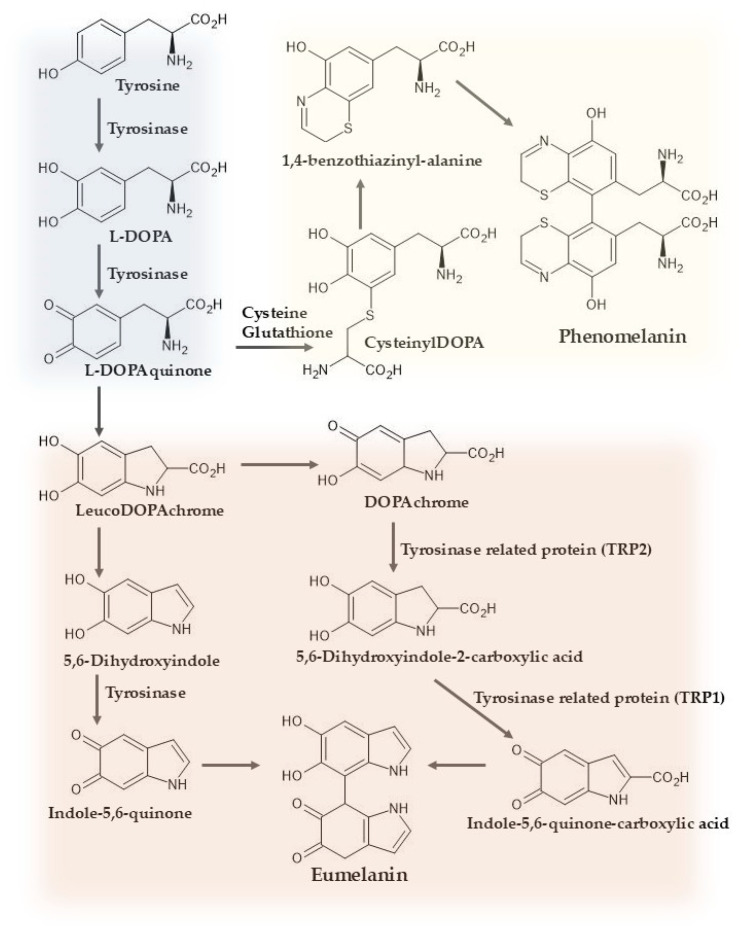
Biosynthetic pathway of eumelanin and pheomelanin from the precursor tyrosine.

Within melanosomes, melanin synthesis is initiated by the TYR-mediated hydroxylation of tyrosine to L-dihydroxyphenylalanine (L-DOPA), followed by oxidation to L-dopaquinone (DQ). DQ functions as a central metabolic branching intermediate that directs the biosynthetic pathway toward either eumelanin or pheomelanin depending on cysteine availability. When the ratio of DQ to cysteine is elevated, DQ undergoes spontaneous intramolecular cyclization followed by oxidation to generate DOPAchrome (DC), a process that predominantly drives the formation of brown-to-black eumelanin [23]. Conversely, cysteine-rich melanosomal conditions favor the production of yellow-to-red pheomelanin [24]. The cystine required for pheomelanogenesis is transported into the cytosol by the xCT transporter encoded by SLC7A11 [25], and then reduced to cysteine by cystine reductase. Increased xCT/SLC7A11 expression enhances the intracellular cystine availability and shifts melanogenic toward pheomelanin [26]. The produced cysteine is subsequently into melanosomes by a major facilitator superfamily domain containing 12 (MFSD12), where it reacts with DQ to generate cysteinyldopa intermediates. These intermediates act as the immediate precursors of pheomelanin and undergo further oxidative reactions within the melanosomal lumen, ultimately yielding mature pigment [27]. The biosynthetic pathway of eumelanin and pheomelanin from the precursor tyrosine is illustrated in Figure 1.

## 3. Melanogenesis and Its Biological Significance

Melanogenesis is a fundamental physiological process responsible for generating melanin pigments that determine cutaneous and hair pigmentation while supporting epidermal homeostasis. This process is primarily regulated by tyrosinase, the rate-limiting enzyme in melanin biosynthesis, whose catalytic activity is controlled by genetic polymorphisms, hormonal regulation, and environmental stimuli such as ultraviolet radiation [28,29]. Upon UV exposure, melanocytes are activated to increase melanin production, and the newly synthesized pigment absorbs and dissipates UV energy. Through this photoprotective function, melanin reduces oxidative stress, preserves genomic integrity, and limits the cellular damage that contributes to photoaging and malignant transformation.

From an evolutionary perspective, variations in constitutive pigmentation reflect adaptive responses to regional solar environments [30,31]. Populations inhabiting UV-intense regions developed higher eumelanin levels that afford enhanced resistance to UV-induced cytotoxicity and lower the risk of sunburn and skin cancer. The variation in pigmentation reflects evolutionary adaptations to different solar environments. In regions exposed to high levels of UV radiation, such as areas near the equator, elevated eumelanin levels produce darker skin tones that enhance UV protection and lower the risk of sunburn and skin cancer [30,31]. In contrast, populations residing in northern latitudes with limited UV exposure display lower levels of melanin synthesis, and an evolutionary adaptation that enhances cutaneous vitamin D production critical for maintaining calcium balance and supporting skeletal health [30,31].

Beyond its photoprotective role, melanin contributes to broader biological functions. The pigment scavenges free radicals and reactive oxygen species, mitigating oxidative stress and inflammation and thereby reducing the likelihood of oxidative stress-related pathological conditions [28]. Melanin also participates in immune modulation and can interact with immune cells, bind microbial components, and influence host defense mechanisms [32].

**Figure 2:**
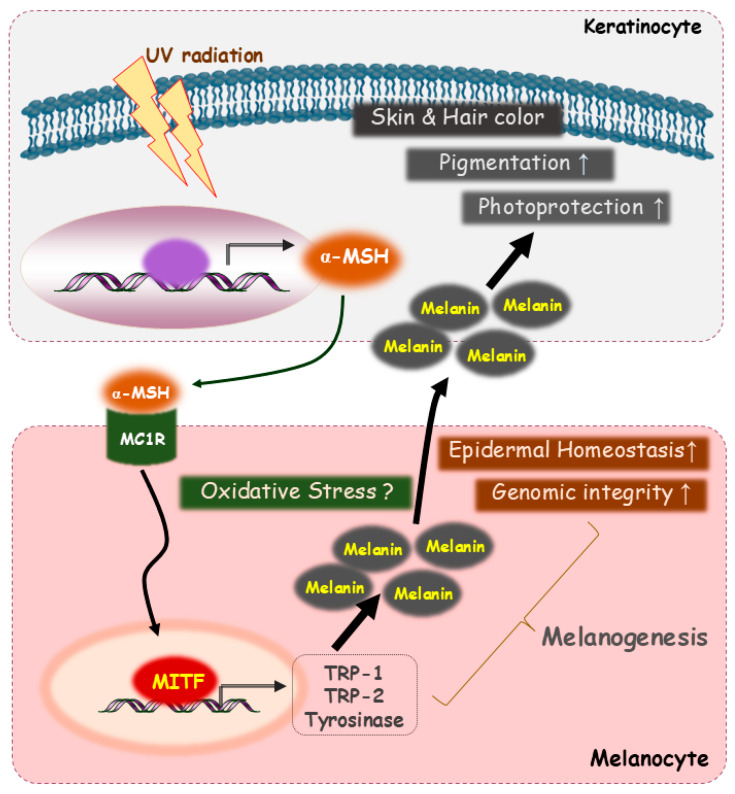
Intercellular signaling between keratinocytes and melanocytes linking UV-responsive α-MSH release to melanogenesis and epidermal protection. UV radiation stimulates keratinocytes to produce and release α-MSH, which binds to MC1R on neighboring melanocytes and enhances the MITF-dependent transcription of melanogenic enzymes, including tyrosinase, TRP-1, and TRP-2. Increased melanin synthesis transfer to keratinocytes contributes to pigmentation, photoprotection, epidermal homeostasis, and genomic integrity. Oxidative stress functions as an additional regulatory cue that influences melanocyte responses and modulates the extent of melanin production within the epidermal environment.

Disruptions in melanin synthesis or distribution underlie numerous pigmentary disorders. Albinism results from genetic defects that impair melanin formation, producing markedly reduced pigmentation and heightened UV sensitivity. Other hypo-pigmentary conditions arise from reduced melanin production or impaired melanosome function, resulting in localized or generalized lightening of the skin [33,34,35]. Conversely, hyper-pigmentary disorders including melasma, solar lentigines, and post-inflammatory hyperpigmentation, emerge from excessive melanogenesis or abnormal melanosome transfer, which are often exacerbated by UV exposure, hormonal changes, or inflammatory stimuli. Together, these conditions highlight the necessity of precise control over melanogenic pathways to preserve cutaneous homeostasis. The intercellular signaling between keratinocytes and melanocytes that links UV-responsive α-MSH release to melanogenesis and epidermal protection is illustrated in Figure 2.

## 4. Anti-Melanogenic Potential of Natural Extracts

Regulation of skin pigmentation has become a major area of interest across dermatological and cosmetic research, driving extensive efforts to identify agents capable of either enhancing or reducing melanin production. In particular, skin-brightening compounds are actively explored not only to improve overall complexion but also to manage hyperpigmentation disorders such as melasma, solar lentigines, and post-inflammatory dark spots, thereby promoting a more uniform and radiant skin tone [36].

Several tyrosinase-targeting agents including hydroquinone, arbutin, kojic acid, and azelaic acid are widely incorporated into cosmetic and therapeutic formulations for hyperpigmentation management [37]. Hydroquinone remains one of the most potent inhibitors through direct interaction with tyrosinase; however, its application is constrained by risks of skin irritation, allergic reactions, and concerns regarding long-term toxicity [37]. Arbutin, a glycosylated derivative of hydroquinone, provides a safer profile with reduced adverse effects, although it often requires higher concentrations to achieve comparable outcomes [38]. Kojic acid, a fungal metabolite, decreases tyrosinase activity by chelating its copper cofactors and is valued for its natural origin, yet challenges related to instability and skin sensitivity remain [39]. Azelaic acid serves as a non-competitive tyrosinase inhibitor with moderate efficacy but may induce discomfort such as burning, stinging, and erythema [40,41].

Although direct tyrosinase inhibitors are commonly employed as long-term skin-whitening agents, their extended use can induce adverse reactions such as redness, itching, tingling, and uneven pigmentation [42,43]. Such limitations highlight the need for alternative approaches with improved safety, physicochemical stability, and pharmacological efficacy. These concerns have accelerated the search for next-generation modulators, especially natural product-derived compounds, that provide more favorable toxicological profiles while enabling more effective regulation of melanogenesis. Naturally occurring compounds that suppress MITF expression or modulate upstream signaling pathways have been characterized as indirect regulators of melanin synthesis. This review highlights fruit-derived natural products and ginsenosides, both of which are extensively utilized in cosmetic formulations, as representative anti-melanogenic candidates with the potential to overcome the limitations of conventional whitening agents.

### 4.1. Grape

Resveratrol is a naturally occurring polyphenolic phytoalexin abundant in grapes, red wine, and berries, and is well recognized for its antioxidant, anti-cancer, anti-inflammatory, and anti-aging properties [44,45,46,47]. In the context of melanogenesis, resveratrol exerts anti-pigmentary activity by suppressing the expression and catalytic function of tyrosinase, TRP-1 and TRP-2, establishing its potential as a skin-brightening agent [45,48,49]. Functionally, resveratrol serves as a competitive tyrosinase substrate producing *o*-quinone species that rapidly oligomerize and may display pro-oxidant properties [50]. The compound exists as *cis* and *trans* isomers, with the trans configuration exhibiting greater stability and superior biological activity. However, UV radiation readily converts trans-resveratrol to its less stable and less active *cis* isomer, raising important considerations for maintaining potency in topical formulations [48,51]. In addition, resveratrol downregulates COX-2 expression in melanocytes and concomitantly reduces MITF and tyrosinase levels. Its regulatory effects involve activation of the ERK1/2 and the PI3K/Akt pathways, and pharmacologic inhibition of either pathway with PD98059 or LY294002 reverses resveratrol-induced suppression of tyrosinase and the MITF, indicating that COX-2-dependent melanogenic control is mediated through these signaling cascades [52].

Oxyresveratrol, a hydroxylated derivative of resveratrol bearing a 2,3,5,4′-tetrahydroxystilbene scaffold, exhibits potent anti-melanogenic effects primarily by inhibiting tyrosinase activity [53]. Both resveratrol and oxyresveratrol have been shown to suppress pigmentation largely through direct binding to and inhibition of tyrosinase [54]. Recent findings demonstrate that oxyresveratrol effectively decreases tyrosinase function and melanin synthesis in B16F10 melanoma cells [55]. Beyond enzymatic inhibition, oxyresveratrol attenuates the MC1R-cAMP-MITF signaling axis and disrupts melanocyte dendrite formation by reducing the expression of small GTPases and kinesins involved in dendrite extension and melanosome transport, including CDC42, RAB11B, RAB17, and RAC1, without altering their cellular structure [55]. These results indicate that oxyresveratrol interferes with melanin synthesis and melanosome transfer, supporting its potential as a therapeutic candidate for treating hyperpigmentation and melanoma-associated pigment dysregulation.

Dihydroresveratrol, produced by hydrogenation of the central double bond of trans-resveratrol or generated from amide-linked derivatives, similarly reduces melanogenesis by suppressing tyrosinase, TRP-1, and TRP-2 activities [56]. In addition to directly inhibiting melanogenic enzymes, it suppresses critical regulator factors such as CREB, phosphorylated CREB, MITF, and TYR, and demonstrates a strong in silico binding affinity for CREB, together supporting its robust anti-melanogenic activity [57]. Notably, although dihydroresveratrol lowers protein levels of MITF, TYR, and TRP-1, it does not alter their mRNA expression, suggesting post-transcriptional or post-translational regulation, potentially via modulation of protein stability or degradation [57]. Structural elements such as carbonyl or amine linkages between phenolic rings may contribute to the differential biological activities observed among resveratrol derivatives [57].

Resveratrol triglycolate, an ester formed by conjugating resveratrol with glycolic acid, has also demonstrated skin-lightening efficacy. In a controlled UV-induced pigmentation study, twice-daily application of a 0.4% resveratrol-triglycolate formulation for up to eight weeks resulted in significantly greater reductions in pigmentation compared with the control group, as assessed by visual grading and instrumental measurements of melanin index, skin lightness, and individual typology angle [58]. Collectively, these findings demonstrate that resveratrol and its derivatives constitute a diverse class of anti-melanogenic agents that act through direct inhibition of tyrosinase and modulation of key melanogenic signaling pathways. These complementary mechanisms highlight their considerable potential as multifunctional candidates for the management of hyperpigmentation and pigment-related disorders.

### 4.2. Mulberry

Mulberry (*Morus alba*) fruit extracts, particularly those obtained from white mulberry, are widely recognized for their potent antioxidant properties, which are attributed to high levels of anthocyanins, quercetin, rutin, and phenolic acids such as gallic and chlorogenic acids [59]. These constituents effectively neutralize reactive oxygen species by donating hydrogen atoms or electrons, thereby reducing the oxidative stress associated with cutaneous aging and inflammation [59]. Prasawang et al. demonstrated that mulberry crude extract (MCE) suppresses UVB-induced melanogenesis in B16F10 melanoma cells and zebrafish embryos [60]. In zebrafish, MCE decreased both the number and spatial distribution of melanocytes, lightened pigmentation, and induced morphological alterations [60]. Consistent with various findings on mulberry-derived bioactive compounds, MCE markedly suppressed UVB-induced tyrosinase activity and melanin production in B16F10 cells [61,62,63]. In addition, 70% ethanol extract of *M. alba* leaves attenuated α-MSH-induced melanin production by downregulating intracellular tyrosinase activity through modulation of the CREB and p38 signaling pathways. Moreover, anthocyanins abundant in mulberry fruit, particularly cyanidin-3-glucoside, further contribute to anti-melanogenic activity by suppressing UVB-induced MITF, tyrosinase, and other melanogenic genes in keratinocytes and melanocytes [64].

Extracts from *M. alba* roots (MAR) have also been shown to diminish intracellular tyrosinase activity without impairing B16F10 cell viability, leading to reduced melanin accumulation. MAR downregulated MITF expression via activation of the ERK pathway and enhanced intracellular sphingosine-1-phosphate (S1P) levels by inhibiting S1P lyase [65]. Increased S1P promotes MITF phosphorylation and degradation, subsequently decreasing the expression of tyrosinase and TRP-1 [66]. Through this S1P-ERK-MITF axis, MAR exerts a multi-level suppression of melanogenesis [65].

Maclurin, a naturally occurring benzophenone derivative found in *M. alba* and mulberry twigs, also contributes to the anti-melanogenic profile of mulberry [67,68]. Structurally, maclurin forms multiple hydrogen bonds, hydrophobic contacts, and aromatic interactions with tyrosinase, enabling strong direct inhibition of the enzyme [69]. Maclurin reduces UVB-induced melanogenesis through enzyme-targeted interactions rather than transcriptional regulation, as it does not alter mRNA expression of tyrosinase, TRP-1, TRP-2, CREB, or MITF [69]. Its anti-melanogenic activity is likely driven by the combined contribution of its antioxidant properties and its direct, binding-mediated inhibition of tyrosinase.

### 4.3. Tropical Fruit Tree

*Garcinia atroviridis*, a tropical fruit tree belonging to Guttiferae family, is naturally distributed across Thailand, Myanmar, and the Indian Peninsula. Its fruits are rich in organic acids, including ascorbic, citric, malic, and tartaric acids [70]. Extracts derived from *G. atroviridis* have been reported to exhibit a broad spectrum of biological activities, notably antimicrobial, antioxidant, and antitumor properties [71]. Among these preparations, the ethanolic fruit extract has gained particular attention for its bioactive composition and regulatory effects on melanogenesis [72]. In α-MSH-stimulated B16F10 cells, this extract reduced intracellular tyrosinase activity and melanin content while downregulating TYR and TRP-1 protein expression. These changes are linked to the suppression of CREB, phosphorylated CREB, and MITF, critical transcriptional mediators of melanogenic signaling [72].

### 4.4. Citrus junos

*Citrus junos* Siebold ex. Tanaka (Rutaceae), known as yuja in Korea, is a citrus species bearing edible yellow fruits that has been reported to reduce oxidative stress and inflammation [73]. Extracts derived from *C. junos* callus extract display marked anti-tyrosinase activity, significantly suppressing DOPA oxidation and melanin biosynthesis [74]. Treatment with the callus extract also promotes fibroblast proliferation and procollagen synthesis. In addition, nanoliposomes loaded with *C. junos* extract have been proposed as anti-aging cosmeceuticals with skin-lightening potential [74]. 4-Hydroxycinnamoyl-malate (4-HCM), a water-soluble constituent of *C. junos* callus, was evaluated for its effects on skin pigmentation by assessing anti-tyrosinase activity and melanogenesis in melanoma cells. To improve cutaneous delivery, 4-HCM was incorporated into elastic nanoliposomes (ENLs) and applied topically to enhance percutaneous absorption. ENL-based formulations achieved an increase in epidermal permeation of 4-HCM, suggesting that inclusion of 4-HCM-loaded ENLs in cosmetic products may offer skin-lightening efficacy [75].

### 4.5. Others

Loquat (*Eriobotrya japonica* Lindl.), an evergreen fruit tree of the Rosaceae family originating from China, is cultivated extensively across subtropical regions [76]. Loquat flowers are rich in phenolic compounds and triterpenes [7,8], which constitute major phyto-nutritional components and contribute to their antioxidant, anti-inflammatory, and anti-diabetic effects [77,78]. These flower extracts inhibit tyrosinase activity in vitro through an anti-competitive mode of action that does not involve copper chelation but instead induces alterations in secondary structures and conformational stability [79]. In addition, the extracts reduce melanin synthesis by downregulating the expression of TYR, TRP1, and TRP2 [79]. Moreover, five flavonoid glycosides isolated from the dried mature seeds of *Ziziphus jujuba* var. inermis (Bunge) Rehder have also demonstrated potent anti-melanogenic activity [80]. Among these, jujuboside A, epicanthic acid, and 6′′′-feruloylspinosin markedly suppressed melanogenesis by inhibiting tyrosinase activity and attenuating the cAMP-CREB-MITF signaling axis [80]. Furthermore, wampee fruit pectin, composing of various monosaccharides including key constituents such as mannose, rhamnose, and galacturonic acid, has been shown to inhibit α-MSH-induced melanogenesis in A375 melanoma cells by modulating the α-MSH-TYR regulatory pathway [81]. Taken together, these compounds underscore the therapeutic promise of structurally diverse botanical metabolites as safe and versatile regulators of melanogenesis, reinforcing their value for the development of pigmentation-modulating agents and skin-brightening formulations.

## 5. Anti-Melanogenic Potential of Ginseng as a Cosmetic Ingredient

*Panax ginseng*, a medicinal species of the Araliaceae family, has been used extensively in East Asian traditional medicine, particularly in Korea and China, for promoting health and managing diseases [82,83]. Its pharmacological actions arise primarily from ginsenosides, a structurally diverse group of triterpenoid saponins abundant in the roots [84]. Beyond traditional applications, ginsenosides exert wide-ranging biological activities, including potent antioxidant and anti-inflammatory effects, contributing to their ability to modulate chronic disease processes and maintain cellular homeostasis [85]. Ginseng root extract has long been utilized in cosmetic formulations due to its anti-melanogenic, anti-aging, anti-wrinkle, and moisturizing activities [86]. It has been reported to enhance antioxidant enzyme activity and suppress ROS generation in skin cells [87,88]. By reducing oxidative stress and directly modulating melanogenic signaling, ginseng extract exerts substantial anti-melanogenic effects. It also promotes collagen synthesis and contributes to wrinkle reduction [89,90,91]. Among various bioactive molecules, ginsenoside Re, which contains a steroid-like aglycone coupled with glycosyl residues, exhibits distinctive anti-melanogenic properties [92]. Re suppresses pigment formation by inhibiting tyrosinase activity as well as its transcriptional regulation. It downregulates MITF and downstream melanogenic enzymes, including tyrosinase, TRP-1, and TRP-2, thereby attenuating pigment synthesis. In addition, ginsenoside Re compound reduces the proliferation of B16F10 melanoma cells and promotes apoptosis, suggesting a potential therapeutic role in melanoma progression [93].

Ginsenoside Rf also modulates melanogenic signaling pathways in B16F10 cells [94]. Rf treatment decreases intracellular concentrations of cAMP, NO, and cGMP, accompanied by reduced activities of adenylate cyclase (AC), protein kinase A (PKA), guanylate cyclase, and protein kinase G (PKG). These findings indicate that ginsenoside Rf suppresses melanogenesis through inhibition of both AC/cAMP/PKA and NO/GC/cGMP/PKG cascades [94]. Consistent with these signaling effects, ginsenoside Rf reduces melanin production and tyrosinase activity in B16F10 cells and zebrafish embryos, accompanied by downregulation of MITF and melanogenic enzymes [94].

The roots of *Panax ginseng* contain several phenolic acids, including salicylic acid (SA), protocatechuic acid (PA), *p*-coumaric acid (*p*-CA), and vanillic acid (VA) which contribute to their biological activities [95]. These compounds significantly reduced melanin content and tyrosinase activity in α-MSH–stimulated B16F10 cells [96]. While α-MSH markedly enhanced TYR, TYRP1, and TYRP2 expression, SA, PA, *p*-CA, and VA effectively attenuated these increases by suppressing MITF expression and inhibiting CREB phosphorylation, thereby targeting the MC1R/cAMP/PKA axis that governs MITF-dependent melanogenesis [97].

(+)-Syringaresinol, a lignan-type polyphenolic metabolite characterized by aromatic ether and furofuran motifs, is present in ginseng berry as well as various other plant species [97,98]. As a bioactive component of Korean ginseng berry, (+)-syringaresinol demonstrates notable anti-inflammatory and anti-aging effects, supporting its relevance in cosmetic applications [99]. It modulates FoxO3a signaling and exhibits potent anti-pigmentary and antioxidant activities, suppressing both cytosolic and mitochondrial ROS [100]. This compound inhibited both mRNA and protein levels α-MSH-induced NOX4 expression, suggesting involvement of the NADPH oxidase pathway. Its depigmenting efficacy has also been verified in a near-physiological artificial pigmented epidermis model, MelanodermTM [100].

Yellow ginseng berry, a newly documented cultivar native to Jilin Province, China, represents a genetically distinct variant of the traditionally cultivated red ginseng berry [101]. This cultivar exhibits strong anti-melanogenic activity in B16F10 melanoma cells [102]. It also demonstrates robust antioxidant properties, reducing lipid peroxidation markers such as MDA, enhancing activities of SOD, CAT, and GSH-Px, and lowering intracellular ROS levels, thereby conferring significant protection against UVB-induced oxidative stress [102].

Hydrolysis-derived metabolites of ginsenosides Rb1 and Rd-including gynostapenoside XVII, gynostapenoside LXXV, ginsenoside F2, and ginsenoside CK-also exert notable anti-melanogenic effects [103]. These rare ginsenosides substantially decreased tyrosinase activity, melanin content, and MITF expression. Molecular interaction analyses revealed high binding affinity toward key amino acid residues (Asp10, Gly68) within the tyrosinase active site, supporting their strong enzymatic inhibitory potential [103].

As the primary physiological barrier against the external environment, the skin is particularly susceptible to the deleterious effects of atmospheric particulate matter (PM). It is well established that PM smaller than 2.5 μm (PM2.5) and that ranging from 2.5 to 10 μm (PM10) can penetrate the stratum corneum and subsequently trigger detrimental cutaneous responses [104]. Moon et al. verified that PM10 not only traverses the skin barrier but also perturbs cutaneous physiology, including the regulation of melanogenesis. Using an ex vivo skin explant model, exposure to 10 μM PM10 markedly elevated MITF and tyrosinase expression [105]. Saponin is a mild detergent that selectively permeabilizes the plasma membrane through its strong interaction with cholesterol-rich lipid domains [106]. Saponin treatment (50 μg/mL) markedly reversed this upregulation. In a keratinocyte-melanocyte coculture system, saponins also effectively mitigated PM10-induced elevation in melanin content [105]. Moreover, PM10 exposure led to increased mRNA expression of pro-inflammatory cytokines, including IL-1α, IL-1β, IL-8, and TNF-α. Co-treatment with saponins suppressed these inflammatory responses [105]. Because inflammation enhances melanogenesis, the anti-inflammatory actions of saponins likely play a central role in preventing PM-induced hyperpigmentation [105].

Korean Red Ginseng Oil (KGO), a lipid-rich preparation derived from ginseng, also displays strong anti-melanogenic activity. KGO inhibits mushroom tyrosinase activity and reduces melanin synthesis in B16F10 cells by downregulating the mRNA and protein expression levels of TRP1, TRP2, TYR, and MITF. [107]. In a UVB-induced skin damage mouse model, topical application of 1% KGO ointment resulted in a pronounced decrease in melanin accumulation compared with UVB-exposed controls [107]. Collectively, these findings demonstrate that resveratrol and its derivatives constitute a diverse class of anti-melanogenic agents that act through direct inhibition of tyrosinase and modulation of key melanogenic signaling pathways. These complementary mechanisms highlight their considerable potential as multifunctional candidates for the management of hyperpigmentation and pigment-related disorders.

Improving the bioavailability of minor ginsenosides has been closely associated with advanced delivery strategies, particularly those employing emulsion- or microcapsule-based systems. Stable emulsions encapsulating minor ginsenosides such as Rg3 and compound K can be formulated through microfluidization [108]. Emulsions generated by this technique exhibit enhanced physicochemical properties, including reduced droplet size and increased stability, providing advantages for topical application and sustained release [109]. Recent advances in micro- and nanoscale delivery technologies have focused on enhancing the membrane permeability of ginsenosides through various encapsulation strategies [110]. Approaches such as liposomal encapsulation, polymer-based micro/nanoparticles, and micro/nanoemulsion systems improve interaction with biological membranes and facilitate more efficient transmembrane transport, thereby increasing the overall bioavailability of ginsenosides [110].

## 6. Pro-Melanogenic Potential of Natural Extracts

In melanocytes, intracellular cAMP levels increase when keratinocytes are stimulated by UV radiation or specific chemical compounds and subsequently release mediators such as histamine, α-MSH, and IL-1β. These signaling molecules interact with their respective receptors on the melanocyte surface to initiate downstream responses [111,112]. Among them, α-MSH binds to MC1R, increasing intracellular cAMP and activating PKA, which phosphorylates CREB, a transcription factor that drives MITF expression and promotes melanogenic gene transcription [113]. In addition to cAMP-dependent signaling, stem cell factor (SCF) binds to the membrane receptor c-kit to regulate MAPK activation, including p38, ERK, and JNK, thereby modulating the expression and activity of melanogenic enzymes [19,114]. More recent studies have shown that bone morphogenetic proteins, particularly BMP-4 and BMP-6, also contribute to pigmentation processes. BMP-6 enhances tyrosinase expression in normal melanocytes by promoting the recruitment and activation of MITF at the tyrosinase promoter through the BMP-6/MAPK signaling [115], while BMP-6/Smad signaling facilitates melanin transfer to neighboring cells [116].

Collectively, these findings indicate that melanogenesis is regulated by multiple convergent signaling pathways. Natural compounds capable of modulating these pathways are increasing being identified, highlighting the importance of establishing a research framework that can integrate and expand upon these mechanistic insights. Notably, some naturally derived substances suppress melanogenesis, whereas others enhance pigmentation depending on biological context and experimental conditions. The following sections highlight representative compounds known to undergo pigmentation through distinct biochemical pathways.

As noted in the previous section, resveratrol is known for its anti-proliferative effects in melanoma, primarily through inhibition of ERK and MEK1/2 signaling. In multiple melanoma models, reduced ERK1/2 activation corresponds with diminished cell proliferation [52,54]. However, in HT-144 human melanoma cells, resveratrol suppresses MEK1/2 phosphorylation and enzymatic activity, leading to cellular differentiation and increased melanogenesis [117]. Consistent with these observations, resveratrol elevates tyrosinase activity, enhances CREB phosphorylation, and ultimately increases melanin synthesis in this model [117].

Tangeretin, a polymethoxylated flavone containing chalcone-related structural features defined by an α,β-unsaturated carbonyl bridge linking two aromatic rings, has been associated with diverse biological activities including antiparasitic, antioxidant, anti-inflammatory, antifungal, and neuroprotective effects [118,119,120,121]. Regarding melanogenesis, methoxylated flavonoids such as nobiletin and tangeretin have been reported to enhance pigmentation in B16F10 melanoma cells [122,123]. Tangeretin, in particular, promotes melanogenic responses by upregulating tyrosinase expression through sustained ERK/MAPK activation, with ERK2 identified as the principal mediator of tangeretin-induced pigmentation [122].

5-Demethylnobiletin (5-hydroxy-6,7,8,3′,4′-pentamethoxyflavone), a hydroxylated polymethoxyflavone enriched in *Citrus reticulata* Blanco, is largely derived from aged orange peel extracts through the spontaneous hydrolysis of nobiletin during storage [124]. Experimental studies have demonstrated that 5-demethylnobiletin activates intracellular cAMP signaling, resulting in elevated CREB phosphorylation and enhances MITF transcriptional activity [125]. The PKA inhibitor H89 markedly attenuated its pigmentation-enhancing effects, reducing melanin content and tyrosinase activity, confirming involvement of the cAMP-PKA-CREB axis [125]. H89 also abolishes 5-demethylnobiletin-induced CREB phosphorylation and downregulates melanogenic markers, including MITF, tyrosinase, TRP-1, and TRP-2, as well as melanosome-associated proteins such as myosin Va, melanophilin, and Rab27a [125]. Based on these findings, Chiu et al. proposed that 5-demethylnobiletin may serve as a potential therapeutic agent for hypopigmentation disorders and could be strategically applied in photoprotective formulation or hair pigmentation treatments [125].

**Figure 3:**
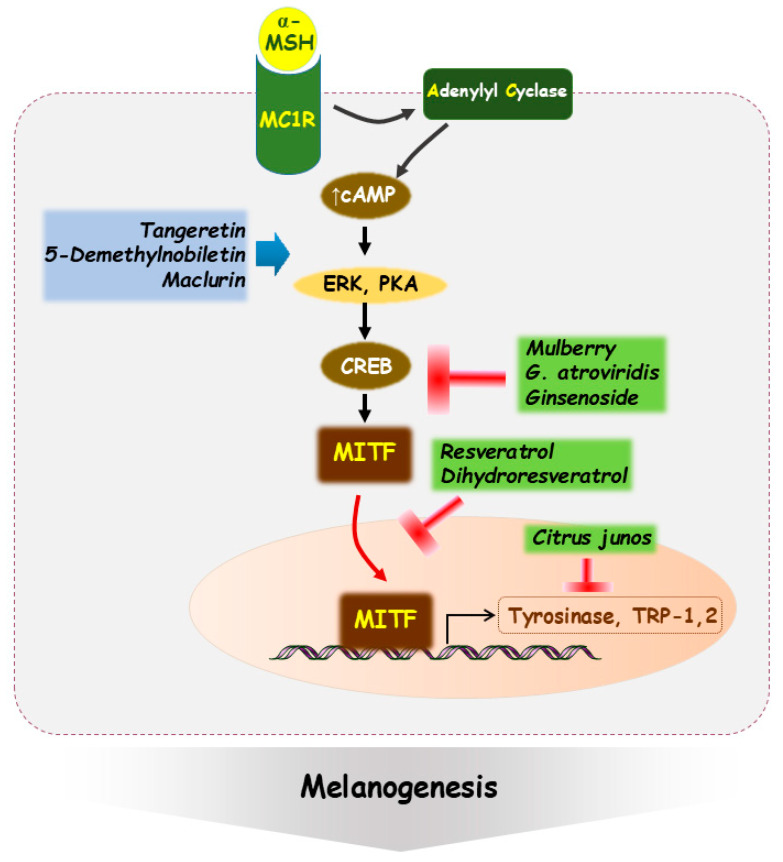
Central regulatory pathway of melanogenesis and its modulation by natural extracts. Binding of α-MSH to MC1R activates adenylyl cyclase, elevating intracellular cAMP levels and stimulating the PKA-dependent phosphorylation of CREB. Activated CREB enhances MITF expression, which promotes the transcription of melanogenic enzymes including tyrosinase, TRP-1, and TRP-2. Natural compounds exert bidirectional influences along this pathway: tangeretin, 5-demethylnobiletin, and maclurin increase melanogenic signaling by sustaining ERK/MAPK or cAMP–PKA–CREB activation, whereas resveratrol, dihydroresveratrol, mulberry extracts, *Garcinia atroviridis*, and *Citrus junos* derivatives attenuate MITF-driven transcription and downstream melanin synthesis.

Maclurin, a phenolic constituent of mulberry twigs, is another pro-melanogenic agent that enhances MITF expression and promotes melanin synthesis. Beyond its pigmentation-inducing effects, maclurin contributes to cellular protection by attenuating H_2_O_2_-induced reductions in cell viability and decreases UVB-induced ROS generation [126]. Collectively, these observations demonstrate that several naturally derived compounds can stimulate melanogenesis through distinct yet convergent mechanisms, primarily involving the activation of ERK/MAPK or cAMP/PKA/CREB signaling pathways, upregulation of MITF and tyrosinase, and, in some cases, simultaneous enhancement of cellular antioxidant defenses, thereby highlighting their potential utility in managing hypopigmentation and supporting pigmentation-related applications [127,128]. Many natural compounds exhibit context-dependent outcomes, with their effects varying according to experimental conditions and concentration. For example, maclurin has been reported to exert anti-melanogenic activity in some studies, whereas other reports indicate the absence of such effects, underscoring the need for additional investigation. Beyond this compound, a broader range of natural substances will require more standardized and systematic research approaches to establish consistent and reliable conclusions. The central regulatory pathway of melanogenesis and its modulation by natural extracts is illustrated in Figure 3.

Melanocytes are subjected to uniquely high oxidative pressure because melanin biosynthesis itself generates ROS. Under continuous exposure to external pro-oxidant stimuli such as UV radiation or chemical compounds, ROS accumulation exceeds the buffering capacity of intrinsic antioxidant defenses and drives oxidative stress. The imbalance disrupts melanocyte physiology, triggering molecular and cellular alterations that promote excessive melanin synthesis, pigment accumulation and, in some cases, pigmentation-related skin disorders [129]. Natural antioxidants provide an opportunity to counteract this imbalance. Vitamin A, and C, along with polyphenols, contribute to ROS neutralization, promote cellular redox homeostasis, and activate repair mechanisms that help maintain melanocyte integrity [130]. By reducing oxidative conditions, these compounds generally contribute to maintaining normal melanocyte function and modulating melanin production. However, reducing ROS levels does not consistently lead to the suppression of melanogenesis. As mentioned above, several studies report that certain natural compounds can attenuate ROS generation while simultaneously activating melanogenic signaling, ultimately increasing melanin synthesis [126]. This indicates that oxidative balance and pigmentation are context-dependent and influenced by compound-specific mechanisms, the cellular metabolic state, and activation of subsequent signaling cascades. Overall, elucidating the regulatory role of oxidative stress in melanocyte biology not only advances the mechanistic understanding of pigmentation disorders, but also indicates that antioxidant-based interventions can exert bidirectional influences on melanin synthesis. A more precise delineation of ROS-dependent and ROS-independent signaling mechanisms is therefore critical for guiding the development of pigmentation-targeted therapeutic approaches that modulate melanin levels without inadvertently exacerbating hyper-pigmentary outcomes. As shown in Table 1, the biological activities and mechanisms of action of currently investigated natural extracts have been comprehensively summarized.

## 7. Limitations of Natural Extracts in Cosmetics

Despite widespread use as plant-based whitening and antioxidant ingredients, natural extracts present several safety and formulation limitations that require careful consideration. The topical application of plant-derived compounds can induce allergic sensitization, irritant reactions, and contact dermatitis, reflecting compositional variability and biochemical complexity [131]. These extracts often contain multiple active and inactive constituents, any of which may elicit skin irritation, sensitization, or photosensitization, highlighting the importance for allergenicity assessment through in vitro assays and human patch testing.

Many plant-derived antioxidants exhibit concentration-dependent pro-oxidant effects, particularly phenolic compounds such as flavonoids and polyphenols that undergo redox cycling or interact with metal ions to generate reactive species [132]. Such pro-oxidant concerns can exacerbate oxidative stress rather than attenuate it. In addition, plant-based whitening agents, including tyrosinase-inhibitory compounds like arbutin, may display cytotoxic effects in melanocytes and keratinocytes at elevated concentrations [133,134]. These concerns are further heightened by the potential structural conversion of arbutin into hydroquinone-related quinones, which raises additional questions about long-term cellular safety. In this regard, the author proposes the need for research that can evaluate the safety of natural products from a structural perspective.

Practical limitations further limit the use of plant-based extracts. Instability during storage, limited dermal penetration, and low bioavailability restrict their functional efficacy in cosmetic formulations. Moreover, higher production costs contribute to increased product pricing compared to synthetic alternatives. Natural product-derived ingredients are governed by heterogeneous regulatory frameworks, with regional variations in permitted concentrations, labeling requirements, and safety documentation.

## 8. Concluding Remarks and Future Perspectives

Melanin plays a fundamental role in maintaining cutaneous homeostasis by providing photoprotection, neutralizing reactive species, and contributing to barrier function and physiological pigmentation balance. Research on naturally derived modulators of melanogenesis has revealed that a diverse range of compounds can either suppress or enhance melanin synthesis through pathways such as ERK/MAPK, cAMP-PKA-CREB, and MITF-mediated transcription, offering promising avenues for the treatment of pigmentation disorders and the development of cosmetic applications. However, despite significant progress, several key challenges remain, including the need for mechanistic clarification across different cellular contexts, evaluation of long-term safety and efficacy, and translation of in vitro findings into clinically meaningful outcomes.

Within this context, fruit-derived extracts offer notable advantages as anti-melanogenic candidates. Their renewable origin, biodegradability, and comparatively lower toxicity align with the increasing demand for sustainable and biocompatible cosmetic actives. In addition, utilizing agriculture-industrial by-products such as fruit peels and seeds, as sources of antioxidant, anti-melanogenic, and preservative compounds, reduces waste while expanding the pool of natural agents for topical applications. Collectively, these features position fruit-origin phytochemicals as promising components of next-generation anti-melanogenic strategies that balance efficacy, safety, and sustainability.

Despite meaningful progress, several critical challenges remain. Differences in underlying regulatory processes across cellular and physiological contexts, limited understanding of long-term safety and efficacy, and the difficulty of translating in vitro findings into clinically meaningful outcomes continue to hinder advancement in this field. Future efforts will require integrated approaches that consider signaling crosstalk, inter-individual variability, and the complex physiological roles of melanin, ensuring that therapeutic modulation does not compromise its essential protective functions.

## Figures and Tables

**Table 1:** Natural extracts and their mechanisms of action.

Natural Extract	Biological Activity	Mechanism of Action
Resveratrol	Melanogenesis ↓	Direct inhibition of tyrosinase, TRP-1, TRP-2 Downregulation of COX-2, MITF Activation of ERK1/2, PI3K/Akt singaling
Oxyresveratrol	Melanin synthesis ↓	Direct binding and inhibition of tyrosinase Reduction in M1CR-cAMP-MITF signaling Suppression of CDC42, RAB11B, RAC1
Dihydroresveratrol	Melanogenesis ↓	Suppression of tyrosinase, TRP-1, TRP-2 Downregulation of CREB, p-CREB, MITF
*Mulberry fruit*	Melanogenesis ↓	Suppression of UVB-induced TYR activity
Mulberry leaf	Melanin synthesis ↓	Downregulation of TYR activity Reduction in CREB and p38 signlaing
Mulberry root	Melanogenesis ↓	Reduction in TYR activity Activation of ERK, downregulation of MITF
Maclurin	Melanogenesis ↓	Reduction in TYR activity Downregulation of CREB, MITF
*Garcinia atroviridis* fruit	Melanin synthesis ↓	Suppression of tyrosinase, TRP-1, TRP-2 Downregulation of CREB, p-CREB, MITF
*Citrus junos* callus	Melanin synthesis ↓	Reduction in TYR activity Suppression of DOPA oxidation
Tangeretin	Pigmentation ↑	Activation of ERK/MAPK Upregulation of TYR expression
5-Demethylnobiletin	Pigmentation ↑	Activation of cAMP/PKA/CREB Elevation of MITF transcriptional activity
Maclurin	Melanogenesis ↑	Upregulation of MITF

The downward arrow indicates decreased activity, whereas the upward arrow indicates increased activity.

## Data Availability

No new data were created or analyzed in this study. Data sharing is not applicable to this article.
